# Hepatitis B virus and HIV co-infection among pregnant women in Rwanda

**DOI:** 10.1186/s12879-017-2714-0

**Published:** 2017-09-11

**Authors:** Mwumvaneza Mutagoma, Helene Balisanga, Samuel S. Malamba, Dieudonné Sebuhoro, Eric Remera, David J. Riedel, Steve Kanters, Sabin Nsanzimana

**Affiliations:** 1 Rwanda Biomedical Center, Ministry of Health, P.O. Box: 7162, Kigali, Rwanda; 2 US Centers for Disease Control and Prevention (CDC), Center for Global Health (CGH), Division of Global HIV/AIDS (DGHA), Kigali, Rwanda; 3 Institute of Human Virology and Division of Infectious Diseases, University of Maryland School of Medicine, Baltimore, MD USA; 4 Global Evaluative Sciences, Vancouver, British Columbia Canada; 5 School of Population and Public Health, University of British Columbia, Vancouver, Canada

**Keywords:** Hepatitis B virus, HIV, Pregnant women, HIV-HBV co-infection

## Abstract

**Background:**

Hepatitis B virus (HBV) affects people worldwide but the local burden especially in pregnant women and their new born babies is unknown. In Rwanda HIV-infected individuals who are also infected with HBV are supposed to be initiated on ART immediately. HBV is easily transmitted from mother to child during delivery. We sought to estimate the prevalence of chronic HBV infection among pregnant women attending ante-natal clinic (ANC) in Rwanda and to determine factors associated with HBV and HIV co-infection.

**Methods:**

This study used a cross-sectional survey, targeting pregnant women in sentinel sites. Pregnant women were tested for hepatitis B surface antigen (HBsAg) and HIV infection. A series of tests were done to ensure high sensitivity. Multivariable logistic regression was used to identify independent predictors of HBV-HIV co-infection among those collected during ANC sentinel surveillance, these included: age, marital status, education level, occupation, residence, pregnancy and syphilis infection.

**Results:**

The prevalence of HBsAg among 13,121 pregnant women was 3.7% (95% CI: 3.4–4.0%) and was similar among different socio-demographic characteristics that were assessed. The proportion of HIV-infection among HBsAg-positive pregnant women was 4.1% [95% CI: 2.5–6.3%]. The prevalence of HBV-HIV co-infection was higher among women aged 15-24 years compared to those women aged 25–49 years [aOR = 6.9 (95% CI: 1.8–27.0)]. Women residing in urban areas seemed having HBV-HIV co-infection compared with women residing in rural areas [aOR = 4.3 (95% CI: 1.2–16.4)]. Women with more than two pregnancies were potentially having the co-infection compared to those with two or less (aOR = 6.9 (95% CI: 1.7–27.8). Women with RPR-positive test were seemed associated with HBV-HIV co-infection (aOR = 24.9 (95% CI: 5.0–122.9).

**Conclusion:**

Chronic HBV infection is a public health problem among pregnant women in Rwanda. Understanding that HBV-HIV co-infection may be more prominent in younger women from urban residences will help inform and strengthen HBV prevention and treatment programmes among HIV-infected pregnant women, which is crucial to this population.

## Background

An estimated 325 million of people, in 2015, were chronic carriers of hepatitis B virus (HBV) or hepatitis C virus (HCV) worldwide [1, 2]. The endemicity is described by the prevalence of the hepatitis B surface antigen (HBsAg) [3]. Globally around 257 million are chronically infected with HBV, and 890,000 die each year from HBV-related hepatic complications [1, 4–6]. Studies have estimated that 30% of cirrhosis and 53% of liver cancer in the world are associated with chronic HBV infection, and 15–40% of patients with chronic HBV infection will develop cirrhosis, liver failure, or hepatocellular carcinoma (HCC) in their lifetime [1, 3]. It is estimated that 70–95% of the adult population in sub-Saharan African countries have been exposed to HBV, and HBsAg sero-prevalence ranges from 6–20% [1–3, 5]. For example, a systematic review and meta-analysis study conducted in Cameroon showed that the overall prevalence of HBV was 11.2% [7], a population-based study of all ages reported an HBsAg prevalence of 17.6% in Northern Uganda which increased with increasing age [8].

HBV is easily transmitted from mother to child, usually at the time of delivery. Without immuno-prophylaxis, the risk of HBV infection among infants born to women who are both HBsAg-positive and HBeAg-positive (a marker of high virus titers) is approximately 85–100%, and 90–95% of these infants become chronically infected with HBV [9, 10]. Knowledge of the prevalence of chronic HBV infection among pregnant women is critical to understand the epidemiology of the disease. Studies and systematic reviews have found that the prevalence of HBsAg among pregnant women in a collection of African nations varies from 2.4–16% [11]. For example, a study conducted among Sudanese pregnant women found a prevalence of 5.6%; [12] similar studies estimated a prevalence of 8.2% in Nigeria [13], 6.3% in Tanzania [14], 8.0% in Mali [15].

HBV and HIV have similarities by exposure to infectious blood and body fluids, but not all the same modes of transmission. Unlike HIV, HBV is not transmitted by breastfeeding, furthermore, child to child transmission is common for HBV but not for HIV and there is evidence suggesting that HBV is more infectious than HIV [2]. There are higher concentrations of HBV in body fluids of persons with acute and chronic HBV infection than among persons with HIV infection. This may possibly explain why HBV might be more infectious than HIV. It is estimated that the prevalence of HBV among HIV infected patients worldwide is 10% and in countries with high HBV prevalence the estimates of HBV infection among HIV infected persons can reach up to 31.3% [16]. Co-infection of HBV and HIV among pregnant women attending ANC in Zambia was 31.3%, 9.0% in Ivory Coast, and 4.9% in Uganda [15]. In North Region of Cameroon, the prevalence of HIV and HBV co-infection was 1.5% [16]. HBV-related liver diseases are more progressive in HIV co-infected patients than in patients with HBV infection alone [1].

Limited information is available on the prevalence of HBV among pregnant women in Rwanda. A study done by Pirillo and colleagues found that the prevalence of HBV among pregnant women in Rwanda was 2.4% [17]. However, that study was not nationally representative. Moreover, a better understanding of factors associated with HBV-HIV co-infection would be beneficial to any prevention programmes aiming to curb the spread of both infections. Therefore, the aim of this study was to estimate the prevalence of chronic HBV infection among pregnant women attending ANC in Rwanda and to determine risk factors associated with HBV and HIV co-infection.

## Methods

### Study population and study design

**Fig. 1:**
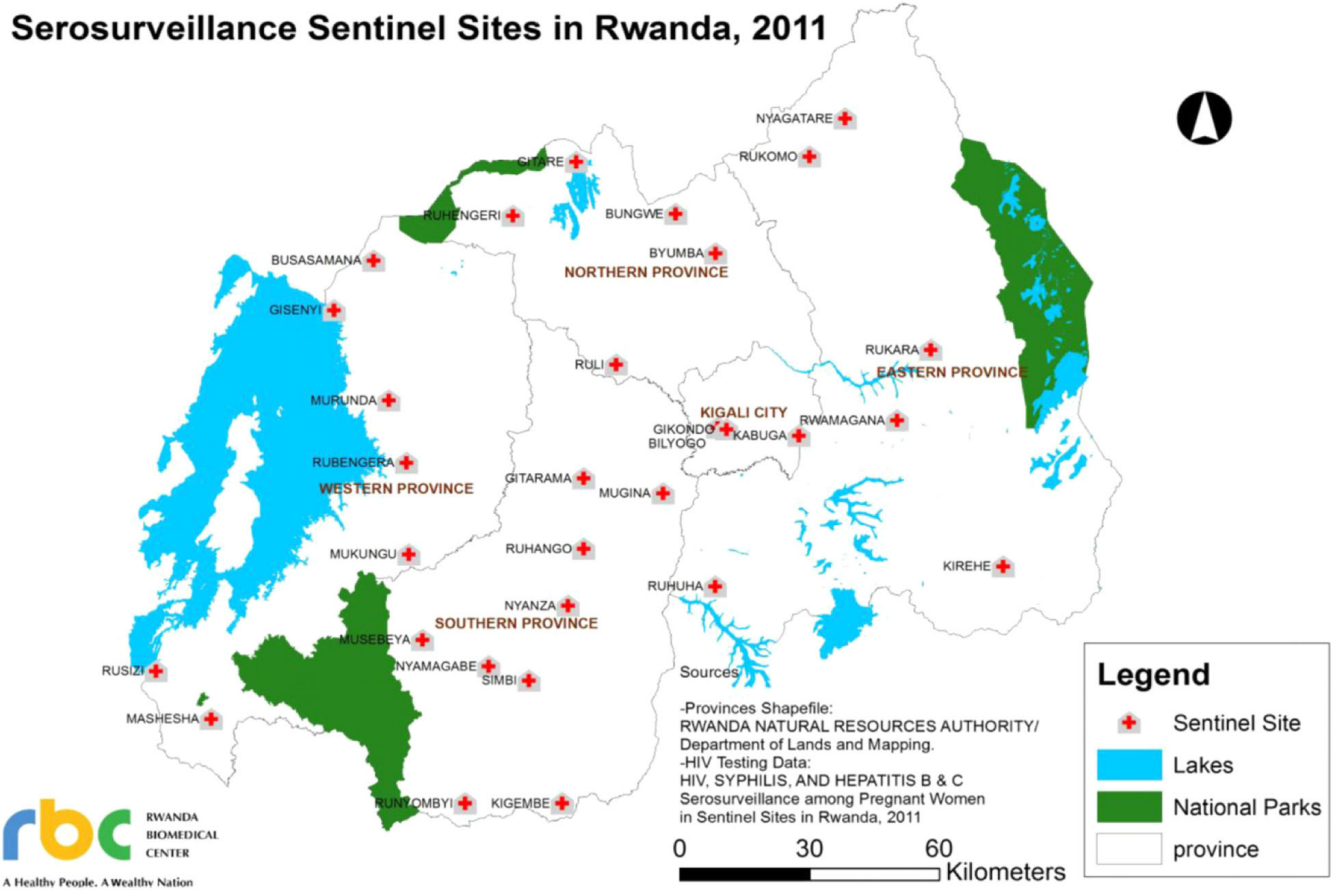
Map of sero-surveillance sentinel sites among pregnant women in Rwanda, 2011. Source: National report of HIV, syphilis, hepatitis B and hepatitis C among pregnant women in Rwanda, 2011

The population consisted of pregnant women attending antenatal clinic and prevention of mother-to-child transmission (ANC-PMTCT) services in 30 sentinel sites national representative in Rwanda in 2011 (Fig. 1). All sentinel sites included in the study and were public health centers. Apart from Kigali City, which was represented by 3 urban sites, each province had at least two urban and two rural sites. Site selection was not randomized for the survey. All 15-49 years pregnant women presenting for their first time at the current pregnancy for ANC-PMTCT services during the surveillance period, able to provide consent, and agreeing to donate venous blood for HBV and HIV tests were included in the study. Women transferred from other health facilities and those coming for follow-up ANC-PMTCT services were not considered in the study.

The Rwanda Biomedical Centre (RBC) maintains a national monitoring and evaluation programme of HIV care services. This was a cross-sectional study, expanding on the RBC surveillance programme through additional surveys, where all women were consecutively enrolled. Each health center has an expected sample size calculated based on the previous HIV prevalence. Once the expected sample size was reached at each health center, the recruitment was stopped. The period of data collection was from May to November 2011. The estimated sample size was 13,267.

### Data collection

A Five-day training was held to cover standard operating procedures following the approved protocol and laboratory techniques related to the surveillance activities. Three local staff members per health center were trained, including two ANC staff and one laboratory technician. A surveillance form was completed for all eligible women who consented to participate. ANC-PMTCT staff administered the form. Blood specimen were collected by venipuncture for routine HIV rapid test and surveillance-related laboratory tests. The specimens were placed in an ethylene-diamine-tetra-acetic acid (EDTA) tube and labeled using the PMTCT code in order to facilitate the return of laboratory test results for clinical care. Blood specimens were centrifuged; plasma was aliquoted into cryotubes at each health center for HIV rapid testing to allow quick clinical care of HIV-positive people. Specimens for surveillance purpose were labeled and stored in a freezer at a temperature of −10° to −20 °C until they were sent to the National Reference Laboratory (NRL) in Kigali. Sentinel sites staff were supervised at least twice monthly by the RBC surveillance staff. Frozen samples were transported by the site supervisors in insulated boxes containing ice packs replenished regularly.

All HBV testing was performed at the National Blood Transfusion Center in Kigali. The testing for HBsAg used the Abbott ARCHITECT system which included reagents, calibrator, control packs, pre-trigger solution and conditioning solution for the assays. Its sensitivity was 99.80% (95% confidence interval 98.90–99.99%) and specificity 100% (95% confidence interval 98.59–100%). HBsAg testing was performed using Architect HBsAg quantitative reagent kit, Architect HBsAg quantitative calibrator and Architect HBsAg quantitative controls. Total HBsAg were tested using the Vitros Chemiluminescence Immuno-assays (Ortho Clinical Diagnostics, Rochester, NY).

Hepatitis B e antigen (HBeAg) testing to detect replication of HBV was applied to HBsAg positive specimens only. This test was performed using ARCHITECT i2000SR, HBeAg Calibrator, control packs, pre-trigger solution and probe conditioning solution for the assays. HBcAb-IgM to detect recent HBV infection (within 6 months) was also applied to HBsAg positive samples only. This test was performed using also ARCHITECT i2000SR, HBcAb-IgM Calibrator, control packs, pre-trigger solution and probe conditioning solution for the assays.

HIV ELISA-based tests were performed at NRL. The screening test used was the HIV Vironostika Uni Form I Ag/Ab, 4th generation. It was in turn confirmed using the Murex HIV Antigen/antibody combination test. All samples with non-reactive results to the screening test were considered negative. Samples reactive to screening test were confirmed by confirmatory test. Samples were considered as definitively positive if they were reactive to both screening and confirmatory tests. If there was any discrepancy between the screening and confirmatory tests, samples were confirmed using Enzygnost and were considered as positive samples if reactive to Enzygnost.

### Data management and analysis

Surveillance forms and laboratory results were double entered using Epi Info 7.0.9.7 (CDC, Atlanta, GA, USA) to minimize errors. To ensure data quality, data verification, and daily back up were performed. All survey data were also backed up on the RBC network server on a daily basis. Data were analyzed using STATA 13 software (StataCorp LP, 4905 Lakeway Drive, College Station, TX, USA). A mixed-effects logistic regression model where the health facility treated as random effects was used to account for the survey design and unknown selection probabilities. Odds ratios were used to measure the magnitude of the effect of HBV in the bivariate analyses of potential risk factors and factors associated with HBV infection at the ≤0.25 significance level were included in the multivariable analysis but kept in the final model only those which were associated at the <0.05 significance level. The following variables were dichotomized as follows age (<25 years and older), marital status (in union or not in union), and number of pregnancies (≤2 or >2). The prevalence of HBsAg and HBeAg among pregnant women was estimated with 95% confidence intervals (95% CI). Model selection for the multivariable model was conducted by minimizing the Akike’s information criterion (AIC) whereby the selected model optimizes both fit and model simplicity. Only HBV and HIV co-infection was examined for associations. The effects of missing values on key variables used in the regression analyses were assessed by fitting indicator variables for missingness for each variable with the outcome; these were tested using maximum likelihood test association with the outcome (missing at random). Missing value records were not included in the analyses because the number of fields and variables with missing data was small. In addition, there was no association between missing data and HIV status. Therefore any bias introduced by this approach will be small.

### Ethical considerations

Blood specimen and interview-based data collection was performed for eligible participants who provided signed informed consent. The protocol was reviewed and approved by the Centers for Disease Control and Prevention and by the Rwandan National Ethics committee. All results were returned to health facilities for appropriate care of patients in case of positive results.

## Results

**Fig. 2:**
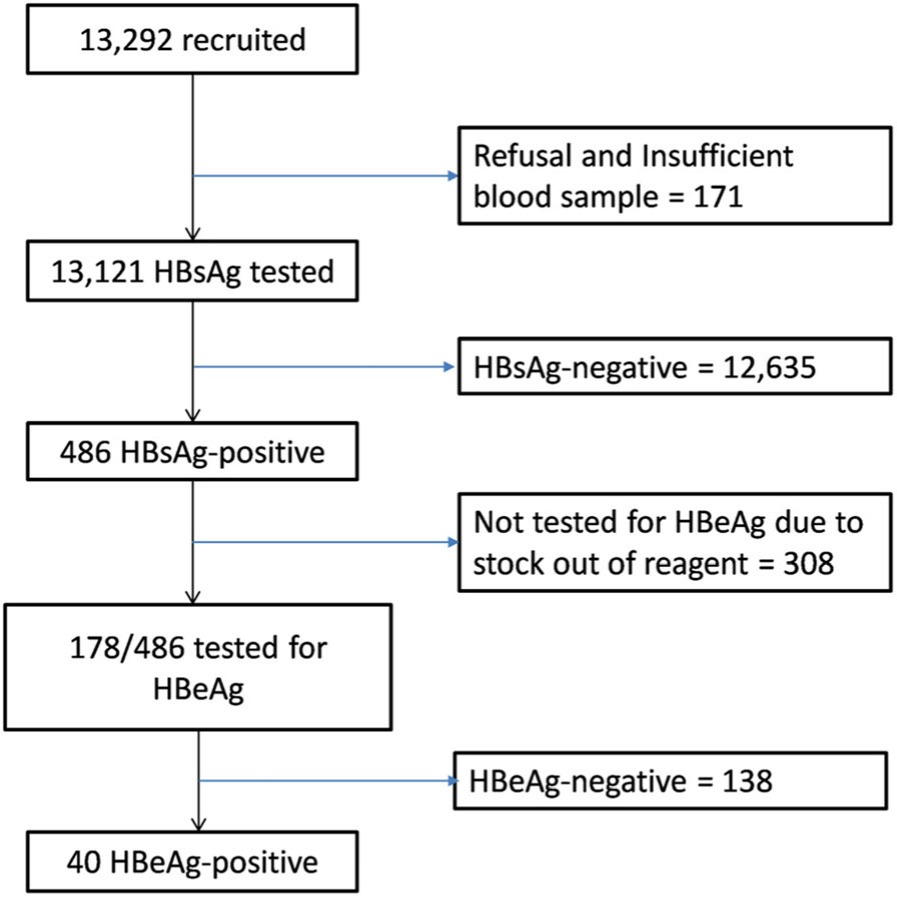
Flow chart of HBV testing among pregnant women in sentinel sites in Rwanda, 2011

Nationally, 13,292 pregnant women were enrolled in the survey and 13,121 of them had HBsAg test results. For different reasons (refusal, insufficient blood sample), 0.6% of participants were not tested for HBsAg. Figure 2 provides further insights into the test result numbers using the flow chart. The overall prevalence of HBsAg was 3.70% (95% CI: 3.8, 4.0%). There were no significant differences in HBsAg prevalence among all socio-demographic characteristics assessed (Table 1). Of the 486 women who tested HBsAg-positive, only 178 (37.0%) samples were tested for HBeAg and 40 samples (22.5%) tested HBeAg-positive. The prevalence of HBcAb-IgM among HBsAg-positive women was 2.1% suggests that there was 2.1 recent HBV infections per every 100 HBsAg positive pregnant women. Of the 486 women who tested HBsAg-positive, 20 women (4.1% [95% CI: 2.5–6.3%]) were also infected with HIV. There was no observed HBV-HIV co-infection among pregnant women in ‘salaried’ occupation, but this co-infection was 4.3% [95% CI: 2.6–6.5%] among pregnant women in ‘not salaried’ occupation. HBV-HIV co-infection was more than three times higher among women in urban areas (6.6% [95% CI: 6.0–7.2%]) compared to women in rural areas (1.9% [95% CI: 0.6, 4.5%]). For other socio-demographic characteristics, there is no significant difference in HBV-HIV co-infection prevalence (Table 2).

**Table 1:** HBV prevalence among pregnant women in Rwanda

Characteristics	Total	HBsAg Positive			*P*-value
	N	n	%	95% CI	
Overall	13,121	486	3.7	[3.4, 4.0]	
Age group					
15–24 yrs	4719	182	3.9	[3.4, 4.5]	0.528
25–49 yrs	8326	303	3.6	[3.2, 4.0]	
Marital Status					
In union	11,366	424	3.7	[3.4, 4.1]	0.993
Not in union	1664	62	3.7	[2.9,4.8]	
Education level					
Illiterate/primary	11,536	425	3.7	[3.4, 4.1]	0.517
Secondary/University	1466	59	4.0	[3.1, 5.2]	
Occupation					
Salaried	500	15	3.0	[1.7,4.9]	0.387
Not salaried	12,492	468	3.7	[3.4, 4.0]	
Residence					
Rural	6547	257	3.9	[3.4, 4.4]	0.180
Urban	6574	229	3.5	[3.1, 3.9]	
Pregnancies					
≤ 2 pregnancies	6946	252	3.6	[3.2,4.1]	0.637
> 2 pregnancies	5837	221	3.8	[3.3,4.3]

**Table 2:** Risk factors for HIV co-infection among HBsAg-positive pregnant women in sentinel sites, Rwanda

	HIV infection among HBsAg-positive pregnant women			Bivariate mixed-effect logistic model	Multiple mixed-effects logistic model
		n	%	Odds ratio (95% CI)	Adjusted odds ratio (95% CI)
Overall	486	20	4.1		
Age group					
25–49 years	303	10	3.3	1	1
15–24 years	182	10	5.5	1.82 (0.71 – 4.55)	6.91 (1.77 – 27.01)
Marital status					
In union	424	17	4.0	1	
Not in union	62	3	3.8	1.19 (0.25 – 5.66)	NA
Education level					
Illiterate/primary	425	17	4.0	1	
Secondary/University	59	3	5.1	1.03 (0.27 – 3.91)	NA
Occupation					
Salaried	15	0	0.0		
Not Salaried	468	20	4.3	PFP	NA
Residence					
Rural	257	5	1.9	1	1
Urban	229	15	6.6	3.28 (1.02 – 10.53)	4.34 (1.15 – 16.43)
Pregnancies					
< = 2 pregnancies	252	8	3.2	1	1
> 2 pregnancies	221	11	5.0	1.60 (0.61 – 4.19)	6.88 (1.71 – 27.77)
RPR results					
RPR negative	475	16	3.4	1	1
RPR positive	11	4	36.4	17.28 (3.98 – 74.93)	24.89 (5.04 – 122.89)

*CI* confidence interval, *RPR* rapid plasma reagin

*PFP* predicts failure perfectly

*NA* not applicable refers to factors with p-value ≥0.25 at the bivariate level which were not considered when developing the Multivariable model. The variables listed in this table are the variables that were included in the bivariate analyses

In the multivariable survey logistic regression model, age, marital status, education level, residence, number of pregnancies, and RPR status were considered as potential risk factors for HBV-HIV co-infection. After adjusting for potential confounders, younger women 15–24 years were more likely to have HBV-HIV co-infection compared to older women aged 25–49 years (aOR = 6.9; (95% CI: 1.8–27.0). Women residing in urban areas were more likely to have HBV-HIV co-infection compared with those residing in rural areas (aOR = 4.3; 95% CI: 1.2–16.4). Women with more than two pregnancies were more likely to have HBV-HIV co-infection (aOR = 6.9; 95% CI: 1.7–27.8). Women who tested RPR-positive were more likely to be infected with HBV-HIV co-infection (aOR = 24.9; 95% CI: 5.0–1224.9) (Table 2).

## Discussion

The prevalence of HBsAg among pregnant women was similar in all sentinel sites surveyed, and the overall HBsAg prevalence (3.7%) is lower than the prevalence found in studies conducted in neighboring countries [8]. There was no significant variation in HBsAg prevalence in different socio-demographic characteristics. HBV prevalence was slightly higher among women tested positive for HIV (i.e., HBV-HIV co-infected women). Despite the low number of HBV-HIV co-infections in our sample, our model revealed multiple variables that were statistically distinguishable between co-infected women and those without the co-infection. These variables could provide insights into prevention strategies to be adopted for this population. Most importantly, the prevalence of HBV-HIV co-infection among younger women may highlights the need to maximize prevention of perinatal and early childhood transmission of HBV by achieving and sustaining high hepatitis B vaccination coverage among children, and by introducing universal hepatitis B birth dose vaccination. On the other hand, the low proportion of chronic HBV infections among HIV-infected patients shows that the hepatitis B vaccination strategies should be largely enforced independently of HIV prevention strategies.

Rwanda has a lower HIV prevalence than many of its immediate neighbours [8, 18, 19]. Potential reasons for the success of Rwanda’s HIV healthcare programme include its mature national programme and universal healthcare; however, reason for success also includes non-transferable qualities, such as its population density [20–22]. HIV prevalence follows the same trend HIV/HBV co-infection in association with higher number of pregnancies, not salaried occupation status, residing in urban residence, and syphilis infection.

We found that the proportion of pregnant women with HIV among HBsAg-positive pregnant women was 4.1%. This co-infection proportion was similar to the HBV and HIV co-infection prevalence among pregnant women in other sub-Saharan African countries: in Nigeria it was 4.2% [23], 4.9% in Uganda [8] and 5.3% from one study done in South Africa [24], lower than the co-infection found in Cameroon (1.5%) [16]. The prevalence of HBV and HIV co-infection in our survey was much lower than the HBV and HIV co-infection found among pregnant women in Zambia (31.3%) [2], Ivory Coast (9.0%) [2], and in one city of Ethiopia (19.0%) [25]. but higher than the HBV and HIV co-infection found by one study in South-Africa (3.1%) [15] In addition to age, our study identified areas of residence as a possible factor of the co-infection –suggesting that effort to identify co-infection persons should start in urban areas and expand out into the rural areas. Having more than two pregnancies indicates the perhaps higher sexual activity. Similarly, in Nigerian women having two or above pregnancies was also identified as risk factor associated with HBV and HIV co-infection [11]. Having a positive RPR test in pregnant women indicates also a possible sexual risky behavior for HIV and HBV infections.

Our survey has a variety of strengths and limitations. Among its strengths, there was a very high participation rate (98.7%). The most important limitation to our study was the lack of randomness in the selection of the sites included in the survey, however, all sentinel sites were selected to represent the country. Given the small number of sentinel sites, we preferred matching over factors we felt were important to keep balanced namely location and whether sites were urban. Results from this survey can therefore not be generalized to all pregnant women in the country. At the time of the survey, there was a shortage of HBeAg reagents; many HBsAg positive samples were not tested for HBeAg. It would therefore not be possible to extrapolate the associations between HBeAg results and any of the assessed characteristic to the entire study population. The HIV prevalence in this manuscript was based on EIA screening test and EIA confirmatory test as per the national guidelines at that time. Based on the current knowledge, the use of two-test EIA with an EIA confirmatory test would result in a high level of false-positives due to the highly sensitive nature of the fourth generation EIAs that were used in the survey. Rwanda is a country with a small population and low HIV prevalence, therefore the problem of false-positives was not excessive. An EIA confirmatory test was also used to confirm HIV rapid test. A Western Blot test would have added value as a tie br**eaker.** Further studies with more representative sample size to confirm what was observed and depict more information are needed.

## Conclusions

HBV infection is a public health problem among pregnant women in Rwanda, as is HBV-HIV co-infection. Understanding that HBV and HBV-HIV co-infection may be more prominent in younger women from urban residences will help strengthen HBV prevention, screening and treatment, which is crucial to the surveyed population of pregnant women in Rwanda. HBV screening in all pregnant women attending ANC services is recommended, especially HIV-positive pregnant women to avoid maternal transmission. The implementation of HBV birth dose vaccination in Rwanda could decrease the burden of HBV among youth.

## Abbreviations

AIC: Akike’s information criterion
ANC: Ante-natal care
aOR: Adjusted odds ratio
CDC: US Centers for Disease Control and Prevention
CGH: Center for Global Health
CI: Confidence Interval
EDTA: Ethylene-diamine-tetra-acetic acid
EIA: Enzyme immunoassay
ELISA: Enzyme-linked immunosorbent assay
HBcAb-IgM: Hepatitis B core antibody immunoglobulin M
HBeAg: Hepatitis B envelop antigen
HBsAg: Hepatitis B surface antigen
HBV: Hepatitis B virus
HCC: Hepatocellular carcinoma
HIV: Human Immunodeficiancy Virus
NRL: National Reference Laboratory
OR: Odds ratio
PMTCT: Prevention of mother-to-child transmission
RBC: Rwanda Biomedical Centre
RPR: Rapid plasma reagin
